# Surfactant-Derived Proteins as Markers of Alveolar Membrane Damage in Heart Failure

**DOI:** 10.1371/journal.pone.0115030

**Published:** 2014-12-16

**Authors:** Paola Gargiulo, Cristina Banfi, Stefania Ghilardi, Damiano Magrì, Marta Giovannardi, Alice Bonomi, Elisabetta Salvioni, Elisa Battaia, Pasquale Perrone Filardi, Elena Tremoli, Piergiuseppe Agostoni

**Affiliations:** 1 Institute of Diagnostic and Nuclear Development, Naples, Italy; 2 Centro Cardiologico Monzino, Istituto di Ricovero e Cura a Carattere Scientifico, Milan, Italy; 3 Department of Clinical and Molecular Medicine, La Sapienza university, Rome, Italy; 4 Section of Cardiology, Department of Medicine, University of Verona, Verona, Italy; 5 Department of Advanced Biomedical Sciences, “Federico II” University, Naples, Italy; 6 Department of Pharmacological and Biomolecular Sciences, University of Milan, Milan, Italy; 7 Department of Clinical Sciences and Community Health, University of Milan, Milan, Italy; The Hospital for Sick Children and The University of Toronto, Canada

## Abstract

**Background:**

In heart failure (HF) alveolar-capillary membrane is abnormal. Surfactant-derived proteins (SPs) and plasma receptor for advanced-glycation-end-products (RAGE) have been proposed as lung damage markers.

**Methods:**

Eighty-nine chronic HF and 17 healthy subjects were evaluated by echocardiography, blood parameters, carbon monoxide lung diffusion (DLCO) and cardiopulmonary exercise test. We measured immature SP-B, mature SP-B, SP-A, SP-D and RAGE plasma levels.

**Results:**

Immature SP-B (arbitrary units), mature SP-A (ng/ml) and SP-D (ng/ml), but not mature SP-B (ng/ml) and RAGE (pg/ml) levels, were higher in HF than in controls [immature SP-B: 15.6 (13.1, 75^th^–25^th^ interquartile range) Vs. 11.1 (6.4), p<0.01; SP-A, 29.6 (20.1) Vs. 18.3 (13.5), p = 0.01; SP-D: 125 (90) Vs. 78 (58), p<0.01]. Immature SP-B, SP-A, SP-D and RAGE values were related to DLCO, peak oxygen consumption, ventilatory efficiency, and brain natriuretic peptide (BNP), whereas plasma mature SP-B was not. The DLCO Vs. immature SP-B correlation was the strongest one. At multivariate analysis, RAGE was associated to age and creatinine, SP-A to DLCO and BNP, SP-D to BNP, mature SP-B to DLCO and creatinine, and immature SP-B only but strongly to DLCO.

**Conclusions:**

Immature SP-B is the most reliable biological marker of alveolar-capillary membrane function in HF.

## Introduction

Impairment of respiratory function is part of the chronic heart failure (HF) syndrome, being both lung mechanics and gas exchange altered. [1] At present, lung dysfunction is evaluated by lung mechanics as well as by gas diffusion analysis. Conversely, notwithstanding possible biological markers of lung damage have been proposed, they are rarely used in HF syndrome. Nonetheless, plasma receptor for advanced glycation end products (RAGE) and surfactant-derived proteins (SPs) are among the most frequently tested. RAGE is a member of the immunoglobin superfamily that amplifies the immune and inflammatory response in several pathophysiological conditions, and it is secreted by several tissues. [2], [3] During lung injury, RAGE is secreted in the alveolar space and in the blood, and it has been proposed as a prognostic marker of lung disease. [4] Several SPs are produced by alveolar cells, and each one has a role in surfactant structure and function. [5]–[7] SPs have been used as lung injury markers, including SP-A, B, and D. [7]–[9] Specifically, SP-A has been suggested as a predictor of lung damage produced by smoke or high altitude, [10] SP-D as a predictor of cardiovascular morbidity and mortality on top of classical risk factors [11] as well as a prognostic marker of chronic kidney [12] and lung diseases, [13], [14] while SP-B has been proposed as a biomarker of alveolar capillary barrier damage both in its mature and immature forms [9], [15]–[19].

In all cases, RAGE and SPs have been linked to alveolar capillary membrane damage, but a comparative evaluation among RAGE and the different SPs available as markers of alveolar capillary membrane damage in HF has not been performed yet. We therefore analyzed the correlation between lung diffusion abnormalities, in terms of carbon monoxide total lung diffusion (DLCO), and RAGE and several SPs in a population of chronic stable HF patients and healthy controls, aiming to identify the ones that better correlates with gas diffusion.

## Methods

### Subjects

We studied HF patients in stable clinical conditions and healthy subjects. Patients belong to a group of individuals regularly followed up at our HF unit and were randomly recruited between February 2012 and November 2012, whereas healthy subjects were hospital staff employees or their relatives with gender and age similar to the HF patients.

Study inclusion criteria for HF patients were New York Heart Association functional classes (NYHA) I to IV, echocardiographic evidence of reduced left ventricular systolic function (left ventricular ejection fraction, LVEF, equal or lower than 45%), optimized and individually tailored drug treatment, stable clinical conditions for at least 2 months, capability/willingness to perform a maximal or nearly maximal cardiopulmonary exercise test (CPET). Patients were excluded if they had severe obstructive and/or restrictive lung disease, anemia (hemoglobin <11 g/dL), history and/or documentation of pulmonary embolism, primary valvular heart disease, pulmonary arterial hypertension, pericardial disease, exercise-induced angina, ST changes, severe arrhythmias and significant cerebrovascular, renal, hepatic and hematological disease.

### Study protocol

At enrolment, demographical and clinical data were collected. Before CPET, in both HF and healthy subjects echocardiographic evaluation, natriuretic peptide B (BNP) and blood samples measurements, standard pulmonary function tests, including DLCO, were performed.

BNP test was performed on UniCel-DxI-800 Access immunoassay (Beckman-Coulter, Fullerton, CA) with Triage-Biosite reagent (San Diego, CA, US). Forced expiratory volume in 1 second (FEV_1_) and lung vital capacity (VC) (Vmax 29C, SensorMedics, Yorba Linda, CA, US), and were standardized as percentages of predicted normal values. [20] FEV_1_/VC lower than 60% was considered indicative of a severe obstructive ventilatory defect [21].

DLCO was measured with the single breath flow technique (Vmax229D, Sensor Medics, Yorba Linda, CA, US). [22] DLCO values were corrected for hemoglobin concentration and were expressed as percentage of predicted values [23].

A CPET familiarization test was always performed. [24] CPET was done on an electronically braked cycle-ergometer (Ergometrics-800, SensorMedics, Yorba Linda, CA, US) using a personalized ramp protocol that was chosen aiming at a test duration of 10±2 minutes. [25] The exercise was preceded by 5 minutes of rest gas exchange monitoring and by a 3-minute unloaded pedaling. A 12-lead ECG, blood pressure and heart rate were also recorded, and arterial oxygen saturation was monitored through a pulse oxymeter. CPET were self-terminated by the subjects when they claimed that maximal effort had been achieved. Oxygen consumption (VO_2_), carbon dioxide production (VCO_2_) and ventilation (VE) were measured breath by breath and reported as average over 20 seconds. All tests were executed and evaluated by 2 expert readers blinded to plasma SPs and RAGE values. The anaerobic threshold (AT) was identified by a standard techniques. [26] The relation between VE and VCO_2_ was calculated as the slope of the linear relationship between VE and VCO_2_ from 1 minute after the beginning of loaded exercise to the end of the isocapnic buffering period.

### Specimen handling and assays

Fresh blood (5 mL) was drawn into Vacutainer tubes containing citrate 0.129 mol/L as an anticoagulant. Plasma was immediately prepared by means of centrifugation at 1,500×*g* for 10 minutes at 4°C, divided into aliquots and frozen at −80°C until assayed.

The analysis of the immature forms of SP-B, which is detectable in the 3 predominant forms. with molecular mass ranging from 17 to 42 kDa, was performed by Western blotting on plasma samples, as previously described. [16] Briefly, in order to precisely resolve low-molecular weight proteins, equal amounts of plasma proteins (50 µg) were separated by one dimensional SDS-PAGE on 15% polyacrylamide gels using a Tris-Tricine buffer system in no reducing conditions. [27] The protein concentration was evaluated by the method of Bradford. [28] Gels were electrophoretically transferred to nitrocellulose at 60 V for 2 hours. Immunoblotting on transferred samples was performed as follows: blocking in 5% (weight/volume) non-fat milk in Tris-buffered saline (100 mmol/L Tris-HCl, pH 7.5, 150 mmol/L NaCl) containing 0.1% Tween 20 (TBS-T) for 1 hour at room temperature; overnight incubation at 4°C with primary antibody against SP-B (rabbit anti-human SP-B H300; Santa Cruz Biotechnology, Santa Cruz, CA, US) diluted at 1∶200 in 5% (w/v) non-fat milk in TBS-T; incubation with secondary goat anti-rabbit antibody conjugated to horseradish peroxidase (Bio-Rad, Milan, Italy) at 1∶1,000 for 1 hour. Bands were visualized by enhanced chemiluminescence using the ECL kit (GE Healthcare, Milan, Italy) and acquired by a densitometer (GS800; Bio-Rad). Bands ranging from 42 kDa to 17 kDa detected by ECL were quantified by densitometry of exposed film using image analysis software (QuantityOne version 4.5.2; Bio-Rad, Milan, Italy). Following transfer, membranes were stained with MemCode reversible protein stain (Pierce Biotechnology, Cramlington, UK) according to the manufacturer's instructions to ensure equivalent loading of protein. For each subject, data are reported as the ratio of band volume, after local background subtraction, *versus* the volume of the total proteins loaded and stained with MemCode. The values were also normalized *versus* the band volume of pooled plasma, loaded as control on each gel, and they are expressed as arbitrary units (AU). Inter-assay coefficient of variation was 12.1±2.9%.

The quantitative analysis of the levels of the mature form (8 kDa) of SP-B was performed by an ELISA purchased from Uscn Life Science Inc. (Wuhan, China). Inter-assay and intra-assay coefficient of variation were 11.6±2.1% and 7.9±1.5%, respectively.

Plasma levels of SP-A, SP-D and RAGE were determined using commercially available ELISA kits (BioVendor, Heidelberg, Germany for SP-A and SP-D; R&D Systems, Minneapolis, MN, US for RAGE). Measurements were performed in duplicate and the results were averaged. The intra-assay and inter-assay coefficients of variation for SP-A were <5% and <10%, Limit of Detection (LOD) is 0.16 ng/ml, and cut off level is 1 ng/ml. For SP-D the intra-assay and inter-assay coefficients of variation were <3% and <4%, LOD is 0.01 ng/ml, and cut off level is 1.56 ng/ml. The intra-assay and inter-assay coefficients of variation for RAGE were <6% and <8%, respectively, minimal detectable dose is 4.12 pg/ml, and the cut off level is 78 pg/ml.

### Statistical analysis

Unless otherwise indicated all data are expressed as means ± SD. Data with skewed distribution are given as median and interquartile range (75^th^ percentile–25^th^ percentile). Categorical variables were compared with χ^2^ test. Unpaired t-test or non-parametric Mann-Whitney test were used when appropriate for between-group comparison. One-way analysis of variance [ANOVA] followed by Kruskal Wallis test were used to compare data for the non-normally distributed variables.

Besides between healthy subjects and HF patients, statistical analysis was also performed by subdividing HF patients according to NYHA class (I–II vs III–IV), median DLCO (≥80% vs <80%), median peak VO_2_ (≥15 mL/kg/min and <15 mL/kg/min), median VE vs VCO_2_ slope (≤30 and >30), median LVEF (≤36% and >36%) and median BNP (≤160 pg/ml and >160 pg/ml). Finally, given that it is well-reported a possible confounding role of smoke on the alveolar-capillary membrane [29] we also explored possible difference between smokers and no smokers within the two study groups. Because SPs and RAGE values showed a non-linear distribution, they were transformed into the natural logarithm, and Pearson correlation was used to disclose possible correlations between these proteins and DLCO, peak VO_2_ value and VE/VCO_2_ slope. To avoid distorted estimates of the effect, Pearson’s correlations were limited to the population with HF. A Bootstrap method, [30] consisting in random population re-sampling to create 2,000 samples, was used to compute 95% confidence interval for the difference between correlation coefficients. A p<0.05 was used to define statistical significance. To identify the independent predictors of increased SPs and RAGE levels, we also performed multivariate analysis. All analyses were performed using SAS statistical package v.9.2 (SAS Institute Inc., Cary, NC, US).

## Results

A total of 106 subjects were evaluated: 89 patients with HF (mean age: 65±11 years, sex: 78 M/11F; mean BMI: 27.4±4.3 Kg/m^2^) and 17 healthy subjects (mean age: 63±9 years; sex: 14 M/3F; mean BMI: 25.9±3.4 Kg/m^2^) (p = NS for all). Active smokers were 30/89 and 7/17 in the HF and control group, respectively. HF etiology was cardiomyopathy due to ischemic coronary disease in 50 cases and not-ischemic in 39 cases. Among HF subjects, 44 were in NYHA class I–II and 45 in class III–IV. The LVEF in HF patients was 36±9%; the median BNP was 160 pg/mL (lower quartile: 57 pg/mL; upper quartile: 407 pg/mL). Treatment in patients with HF included angiotensin-converting enzyme inhibitors in 53 cases (60%), AT1 blockers in 21 cases (24%), β-blockers in 81 cases (91%), diuretics in 59 cases (66%), antialdosteronic drugs in 43 cases (48%), amiodarone in 37 cases (42%), digoxin in 4 cases (5%), antiplatelet in 59 cases (66%), and anticoagulant in 24 cases (27%).

HF patients showed a significantly lower VC, FEV_1_ and DLCO than healthy subjects, whereas there was no difference in FEV_1_/VC ratio (Table 1). Exercise performance and ventilatory efficiency were significantly reduced in HF patients (Table 1). Plasma total immature SP-B, SP-A and SP-D levels significantly differed between HF population and healthy subjects, whereas mature SP-B and RAGE levels did not differ between the two groups (Table 1). Between current smokers and no smokers in HF population there were not significant differences of SPs and RAGE levels (data not shown). In healthy subjects, no significant differences of SPs levels were observed, whereas RAGE significant differed between smokers and no smokers, specifically 720 (297) Vs 1260 (637) pg/ml, p = 0.05.

**Table 1:** Pulmonary function, cardiopulmonary exercise (CPET), and laboratory data in the two study groups.

	Heart FailurePatients (n = 89)	Healthysubjects (n = 17)	P values
*Pulmonary function data*			
**VC (L)**	3.62±0.78	4.35±1.05	0.01
**VC (% predicted)**	93±16	109±15	<0.01
**FVC (L)**	3.51±0.80	4.27±1.09	0.01
**FVC (% predicted)**	93±16	110±14	<0.01
**FEV_1_ (L)**	2.56±0.63	3.29±0.89	<0.01
**FEV_1_ (% predicted)**	87±17	104±15	<0.01
**FEV_1_/FVC**	73±9	77±7	NS
**DLCO (mL/mm Hg/min)**	19±5	27±8	<0.01
**DLCO (% predicted)**	73±15	95±20	<0.01
*CPET data*			
**Peak Workload (W)**	85±36	162±61	<0.01
**Peak VO_2_ (mL/min/Kg)**	16±6	30±8	<0.01
**VO_2_ at AT (mL/min/Kg)**	11±3	19±5	<0.01
**VE/VCO_2_ slope**	31±7	25±4	<0.01
*Laboratory data*			
**Immature SPB (AU)**	15.6 (13.1)	11.1 (6.5)	<0.01
**Mature SPB (ng/mL)**	190 (140)	244 (496)	NS
**SPA (ng/mL)**	29.6 (20.1)	18.3 (13.5)	0.01
**SPD (ng/mL)**	125 (88)	78 (38)	<0.01
**RAGE (pg/mL)**	1485 (1139)	1236 (570)	NS

Data are presented as means ± SD or as median (75^th^–25^th^ interquartile range). **AT** = anaerobic threshold; **DLCO** = carbon monoxide lung diffusing capacity; **FEV_1_** = forced expiratory volume in 1 s; **FVC** = forced vital capacity; **NS** = not significant; **VC** = vital capacity; **VCO_2_** = carbon dioxide consumption; **VE** = ventilation; **VO_2_** = oxygen consumption. Surfactant proteins (**SPs**) values were normalized for total proteins. **AU** = arbitrary unit; **RAGE** = plasma receptor for advanced glycation end products.

**Figure 1:**
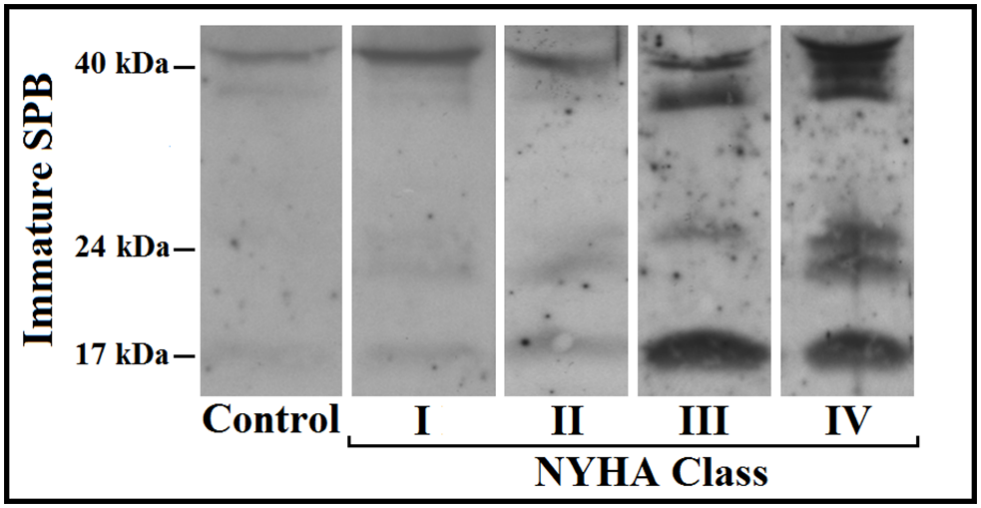
Representative image for immature surfactant protein type B (SPB) immunoblotting of plasma samples derived from the control group and HF patients grouped according New York Heart Association (NYHA) class.

Table 2 shows the detailed comparison between plasma total immature SP-B, mature SP-B, SP-A, SP-D and RAGE levels in HF patients when categorized for HF severity as defined by NYHA class, DLCO, peak VO_2_, VE vs. VCO_2_ slope, and BNP (66 cases) levels. Only immature SP-B and mature SP-A were higher in patients with most severe HF considering all the above reported HF severity criteria (Table 2, and Fig. 1).

**Table 2:** Surfactant proteins and RAGE levels in healthy controls and heart failure patients, categorized according to different disease severity variables.

	Controls	HF-1 Group	HF-2 Group	ANOVA
		NYHA I–II	NYHA III–IV	
	(n = 17)	(n = 44)	(n = 45)	
**Immature SPB (AU)**	11.1 (6.5)*	12.1 (9.8)§	20.4 (18.5)	**<0.01**
**Mature SPB (ng/mL)**	244 (496)	186 (95)	205 (162)	NS
**SPA (ng/mL)**	18.3 (13.5)*	26.7 (17.5)§	33.5 (24.8)	**<0.01**
**SPD (ng/mL)**	78 (38)# *	112 (65)†	142 (121)	**<0.01**
**RAGE (pg/mL)**	1236 (570)	1271 (1127)	1708 (1238)	NS
		**DLCO≥80%**	**DLCO<80%**	
	**(n = 17)**	**(n = 31)**	**(n = 58)**	
**Immature SPB (AU)**	11.1 (6.5)*	10.2 (8.6)§	19.4 (17.3)	**<0.01**
**Mature SPB (ng/mL)**	244 (496)	190 (159)	203 (138)	NS
**SPA (ng/mL)**	18.3 (13.5)*	22.6 (18.9)§	32.9 (23.5)	**<0.01**
**SPD (ng/mL)**	78 (38)*	103 (73)	136 (96)	**<0.01**
**RAGE (pg/mL)**	1236 (570)	1266 (1188)	1520 (1107)	NS
		**Peak VO_2_≥15 mL/Kg**	**Peak VO_2_<15 mL/kg**	
	**(n = 17)**	**(n = 43)**	**(n = 46)**	
**Immature SPB (AU)**	11.1 (6.5)*	11.9 (9.8)§	20.3 (18.3)	**<0.01**
**Mature SPB (ng/mL)**	244 (496)	195 (163)	188 (128)	NS
**SPA (ng/mL)**	18.3 (13.5)*	25.8 (18.6)§	33.1 (24.8)	**<0.01**
**SPD (ng/mL)**	78 (38)# *	112 (75)	133 (103)	**<0.01**
**RAGE (pg/mL)**	1236 (570)	1266 (1228)	1520 (1111)	NS
		**VE/VCO_2_ slope≤30**	**VE/VCO_2_ slope>30**	
	**(n = 17)**	**(n = 51)**	**(n = 38)**	
**Immature SP-B (AU)**	11.1 (6.5)# *	12.8 (13.6)†	18.1 (21.4)	**<0.01**
**Mature SPB (ng/mL)**	244 (496)	200 (150)	180 (144)	NS
**SPA (ng/mL)**	18.3 (13.5)*	26.7 (17.4)§	35.8 (21.1)	**<0.01**
**SPD (ng/mL)**	78 (38)# *	111 (63)§	149 (157)	**<0.01**
**RAGE (pg/mL)**	1236 (570)	1276 (1098)†	1732 (1250)	**0.05**
		**BNP≤160 pg/mL**	**BNP>160 pg/mL**	
	**(n = 17)**	**(n = 30)**	**(n = 36)**	
**Immature SPB (AU)**	11.1 (6.5)*	12.3 (14.1)†	18.7 (18.6)	**<0.01**
**Mature SPB (ng/mL)**	244 (496)	190 (137)	200 (217)	NS
**SPA (ng/mL)**	18.3 (13.5)*	22.8 (17)§	37.2 (19.9)	**<0.01**
**SPD (ng/mL)**	78 (38)*	96 (73)§	137 (147)	**<0.01**
**RAGE (pg/mL)**	1236 (570)*	1243 (1024)§	1893 (1559)	**<0.01**

Data are presented as means ± SD or as median (75^th^–25^th^ interquartile range).

*p<0.01 Healthy subjects Vs HF-1 Group;

# p<0.05 Healthy subjects Vs HF-1 Group;

§ p<0.01 HF-1 Group Vs HF-2 Group;

† p<0.05 HF-1 Group Vs HF-2 Group.

**HF** = heart failure; **LVEF** = left ventricular ejection fraction; **BNP** = brain natriuretic peptide. For other abbreviations see table 1.

We analyzed the association between the different SPs and RAGE levels and clinical, demographic, blood, echocardiographic, pulmonary function test and exercise performance parameters (Table 3). Plasma total immature SP-B, SP-A, SP-D and RAGE values were significantly related to DLCO, peak VO_2_, VE/VCO_2_ slope, and BNP value, whereas plasma mature SP-B was not. When Pearson’s correlation coefficients between plasma total immature SP-B, SP-A, SP-D and RAGE levels vs. DLCO were compared, the Pearson’s coefficient of correlation between plasma total immature SP-B levels vs. DLCO values was significantly higher than the coefficients of correlation between other proteins and DLCO. On this regard no difference was observed for peak VO_2_, VE/VCO_2_ slope, LVEF and BNP (Table 4).

**Table 3:** Relationship between surfactant proteins and RAGE levels and general, clinical, pulmonary function and cardiopulmonary exercise data in the heart failure population.

		Immature SPB	Mature SPB	SPA	SPD	RAGE
**AGE (yrs)**	r value	0.265	0.013	0.256	0.152	0.066
	p value	**0.012**	NS	**0.015**	NS	NS
**BMI (Kg/m^2^)**	r value	–0.156	0.144	–0.052	–0.203	–0.285
	p value	NS	NS	NS	NS	**<0.01**
**DLCO (%pred)**	r value	–0.591	–0.088	–0.429	–0.393	–0.245
	p value	**<0.01**	NS	**<0.01**	**<0.01**	**0.023**
**pVO_2_ (mL/Kg)**	r value	–0.426	–0.012	–0.408	–0.292	–0.265
	p value	**<0.01**	NS	**<0.01**	**<0.01**	**0.01**
**VE/VCO_2_ slope**	r value	0.363	–0.037	0.455	0.333	0.406
	p value	**<0.01**	NS	**<0.01**	**<0.01**	**<0.01**
**BNP (pg/mL)**	r value	0.367	–0.008	0.502	0.412	0.490
	p value	**<0.01**	NS	**<0.01**	**<0.01**	**<0.01**
**Hb (g/dL)**	r value	–0.088	0.067	–0.170	–0.069	–0.285
	p value	NS	NS	NS	NS	**<0.01**
**Creatinine (mg/dL)**	r value	0.268	–0.253	0.245	0.184	0.367
	p value	**<0.01**	**0.016**	**0.02**	NS	**<0.01**

SPs, RAGE, Peak VO_2_, VE/VCO_2_ slope and BNP levels are transformed into natural logarithm. BMI: body mass index. For all abbreviations see table 1 and table 2.

**Table 4:** Comparison between Pearson’s correlation coefficients between plasma immature SP-B, SP-A, SP-D and RAGE levels, pulmonary function and cardiopulmonary exercise data in the heart failure population.

	ImmatureSPB vs. SPA		ImmatureSPB vs. SPD		ImmatureSPB vs. RAGE		SPA vs.SPD		SPA vs.RAGE		SPD vs.RAGE	
	*CI* *95%*	*p* *value*	*CI* *95%*	*p* *value*	*CI* *95%*	*p* *value*	*CI* *95%*	*p* *value*	*CI* *95%*	*p* *value*	*CI* *95%*	*p* *value*
**DLCO** **(%pred)**	(0.154;0.294)	**<0.05**	(–0.008;0.326)	**0.06**	(0.141;0.500)	**<0.05**	(–0.136;0.145)	NS	(–0.317;–0.007)	**<0.05**	(0.014;0.317)	**<0.05**
**Peak VO_2_** **(mL/Kg)**	(–0.224;0.073)	NS	(–0.305;0.038)	NS	(–0.426;–0.050)	**<0.05**	(–0.221;0.109)	NS	(–0.347;0.034)	NS	(–0.272;0.058)	NS
**VE/VCO_2_** **slope**	(–0.078;0204)	NS	(–0.164;0.141)	NS	(–0.227;0.133)	NS	(–0.081;0.212)	NS	(–0.040;0.266)	NS	(–0.192;0.111)	NS
**LVEF** **(%)**	(–0.129;0.205)	NS	(–0.183;0.173)	NS	(–0.328;0.110)	NS	(–0.147;0.217)	NS	(–0.334;0.046)	NS	(–0.064;0.277)	NS
**BNP** **(pg/mL)**	(–0.318;0.060)	NS	(–0.263;0.220)	NS	(–0.276;0.232)	NS	(–0.285;0.088)	NS	(–0.297; 0.090)	NS	(–0.172;0.175)	NS

SPs, RAGE, Peak VO_2_, VE/VCO_2_ slope and BNP levels are transformed into natural logarithm. **BNP** = brain natriuretic petide; **CI** = confidence interval. For other abbreviation see table 1 and 2.

**Figure 2:**
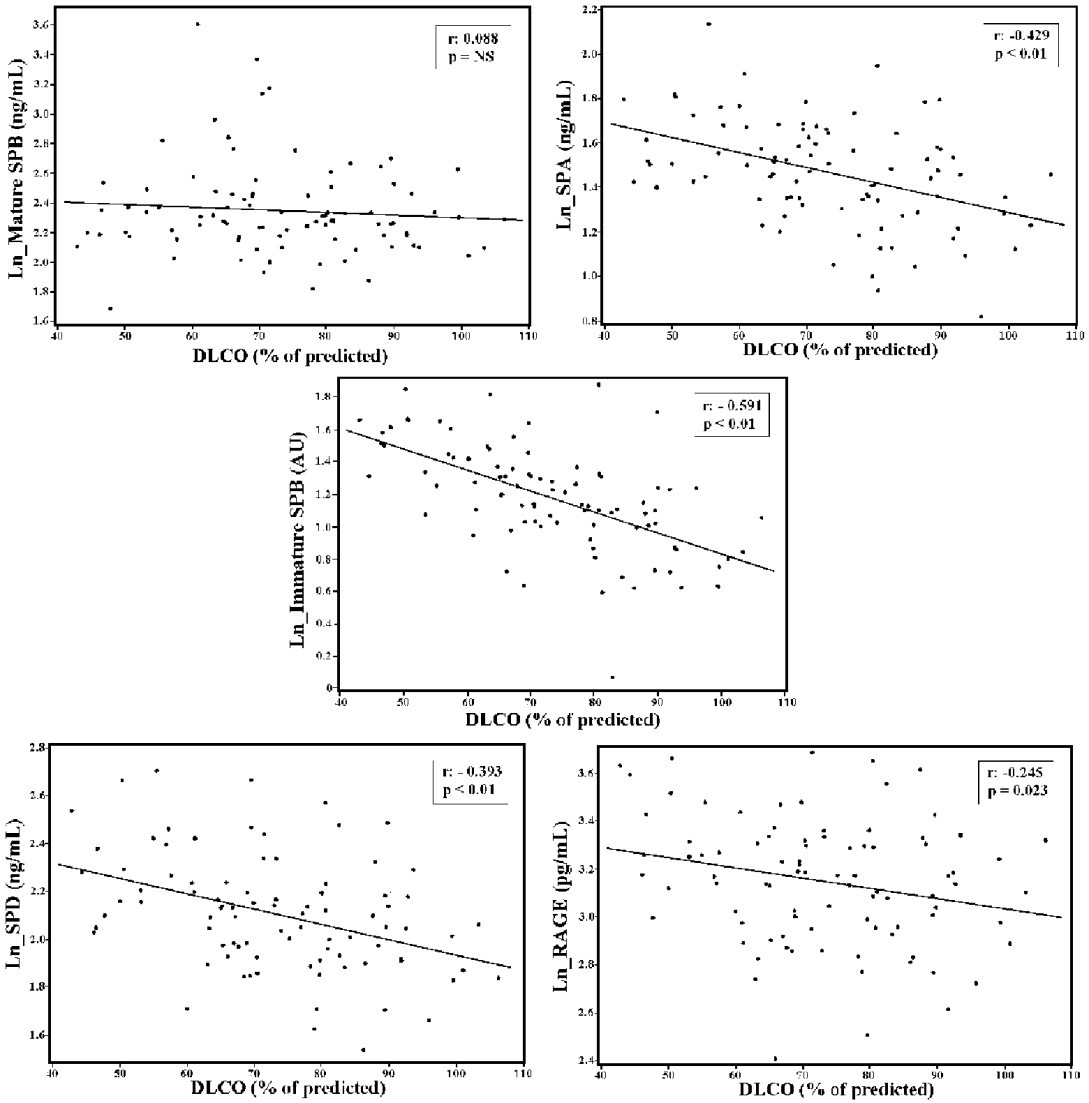
Relationship between each of the five studied serum biomarkers and lung diffusing capacity values in the heart failure population. Immature SP-B levels are transformed into natural logarithm (Ln). DLCO = carbon monoxide lung diffusing capacity corrected for hemoglobin concentration; SP = surfactant protein; RAGE = plasma receptor for advanced glycation end products. Note that the strongest relationship was the one between the Immature SPB and DLCO (see also table 4).

At multivariate analysis, BNP levels were independently associated with SP-A and SP-D while creatinine levels were independently associated with mature SP-B and RAGE. Differently immature SP-B, mature SP-B and SP-A are independent predictors of impaired DLCO, albeit immature SP-B was much stronger (Table 5). The correlation between plasma total immature SP-B levels and DLCO in HF patients is reported in Fig. 2.

**Table 5:** Multiregression analysis for the different SPs and RAGE (dependent variables).

	p value				
	Immature SP-B	Mature SPB	SP-A	SPD	RAGE
**Age (yrs)**	0.88	0.84	0.95	0.37	**0.03**
**BMI (Kg/m^2^)**	0.88	0.97	0.62	0.94	0.21
**DLCO (%)**	**0.001**	**0.05**	**0.05**	0.23	0.76
**Peak VO_2_ (mL/Kg)**	0.70	0.25	0.65	0.64	0.81
**VE/VCO_2_ slope**	0.88	0.97	0.12	0.47	0.16
**LVEF (%)**	0.54	0.35	0.77	0.09	0.45
**BNP (pg/mL)**	0.53	0.15	**0.04**	**0.05**	0.07
**Hemoglobin (mg/dL)**	0.20	0.84	0.85	0.25	0.50
**Creatinine (mg/dL)**	0.87	**<0.01**	0.86	0.78	**0.01**

SPs, RAGE, Peak VO_2_, VE/VCO_2_ slope and BNP levels are transformed into natural logarithm. For abbreviation see table 1 and 2.

Finally, the correlation among the different SPBs studied and RAGE in HF patients is reported in table 6.

**Table 6:** Relationship between surfactant proteins and RAGE levels in the heart failure population.

		Immature SPB	Mature SPB	SPA	SPD
**Immature SPB (AU)**	r value				
	p value				
**Mature SPB (ng/ml)**	r value	0.097			
	p value	NS			
**SPA (ng/ml)**	r value	0.511	0.295		
	p value	**<0.01**	**<0.01**		
**SPD (ng/ml)**	r value	0.470	0.138	0.547	
	p value	**<0.01**	NS	**<0.01**	
**RAGE (pg/ml)**	r value	0.261	0.039	0.511	0.573
	p value	**<0.05**	NS	**<0.01**	**<0.01**

SPs and RAGE levels are transformed into natural logarithm. For abbreviation see table 1 and 2.

## Discussion

In HF alveolar capillary membrane is abnormal. In the present study we analyzed in HF patients which among SPs and RAGE was the most reliable marker of alveolar capillary membrane function, as assessed in term of DLCO. Indeed, the strongest correlation between DLCO, an easy and noninvasive index of alveolar capillary unit function, and the five explored serum biomarkers was observed for immature SP-B. We also observed that immature SP-B, mature SP-A and SP-D, but not RAGE and mature SP-B values, were higher in HF patients than in healthy controls. Moreover, patients with more severe HF – according to NYHA classification, peak VO_2_, VE/VCO_2_ slope or BNP values – all strong HF prognosis predictors – showed higher plasma levels of immature SP-B and SP-A than patients with less severe HF.

Few relevant study limitations should be discussed at first. To start with the results of the present study should be considered only within the frame of the present setting, that is analysis in the blood of markers of possible alveolar capillary membrane dysfunction in chronic HF patients in stable clinical conditions. Indeed, previously, SPs have been also measured in the alveolar lavage fluids a possibility, in clinical practice, unlikely in HF patients. As regards RAGE it should be noticed that, previously, it has been reported to increase in case of acute lung diseases, such as acute lung injury or acute respiratory distress syndrome, as well as during mechanical ventilation or cardiopulmonary bypass. [3], [4], [17], [18] This is not surprising because RAGE is activated during an inflammatory reaction, which is present in patients with stable HF as shown in the general laboratory data analysis. As a matter of facts hemoglobin, kidney function and BNP were all correlated to RAGE confirming also the not-organ specific origin of RAGE increase in HF. As regards SP-B, a significant increase of the mature form of SP-B has been reported after acute pulmonary edema, mechanical ventilation and cardiopulmonary bypass, [9], [17], [18], [31] while a simple hemodynamic stress as that obtainable through exercise in non-cardiac patients, or exposure to high altitude in high altitude pulmonary edema (HAPE)-free subjects is unable to increase the mature form of SP-B, SP-A and RAGE. [10], [32] The bulk of these findings suggests that mature forms of SPs increase only when alveolar cells are severely damaged and not during a simple hemodynamic stress. Indeed, at high altitude, although a limited DLCO reduction has been described in HAPE-free subjects, a permanent damage of the alveolar capillary membrane is unlikely, being DLCO normalized after 2 weeks of high-altitude sojourn and increased if the sojourn is more prolonged. [32] In this context, a further study limitation is that we have studied patients only once and, accordingly, we were unable to analyze whether SPs or RAGE changes correlates with DLCO changes in a single patients. We also acknowledge that we have not reported data for SPC given that we did not detect any immunoreactive signal in the human plasma of both healthy subjects and patients. [33] A final study limitation which we have to acknowledge is that cut-off values for separate patients with severe from moderate HF, such as NYHA, peak VO_2_, VE/VCO_2_ slope, DLCO and BNP were totally arbitrarily and calculated as median value in our HF population.

In HF, the alveolar capillary unit undergoes a remodeling process which, in the long run, generates changes which are independent from the hemodynamic pattern [34], [35] and which could be easily and noninvasively evaluated through the DLCO analysis. Indeed, the DLCO represents a solid index of HF severity, a prognostic index of HF and even a target for HF therapy [1], [36], [37] and it has been demonstrated that the major determinant of its reduction was the alveolar capillary membrane remodeling (i.e. thickening due to fibrosis and cellular proliferation) with an almost unchanged capillary blood flow [1], [36]. Although it should be highlighted that many clinical conditions and, particularly, those characterized by ventilation-perfusion mismatch could alter the DLCO meaning, present data come from a study sample almost free from possible confounders (i.e. we excluded patients with severe obstructive lung disease, pulmonary arterial hypertension, etc.). In this context our data allows us to hypothesize that the underlying mechanism of the observed relationship between DLCO and the immature form of SP-B may be, at least in part, due to an alveolar-capillary membrane damage. Nonetheless we should acknowledge that, at present, we do not know whether a pharmacological change in DLCO is associated with a change in these serum parameters. [37]–[40] Interestingly, plasma levels of all SPs, but the mature form of SP-B, were higher in HF patients with impaired lung diffusion but apparently unrelated to organs damage different from the lung. Therefore, immature SP-B, SP-A and SP-D can be considered markers of alveolar capillary membrane dysfunction. Indeed they all correlate between each other (table 6). Differently mature SP-B seems to increase only in case of acute alveolar capillary membrane injury [15].

**Figure 3:**
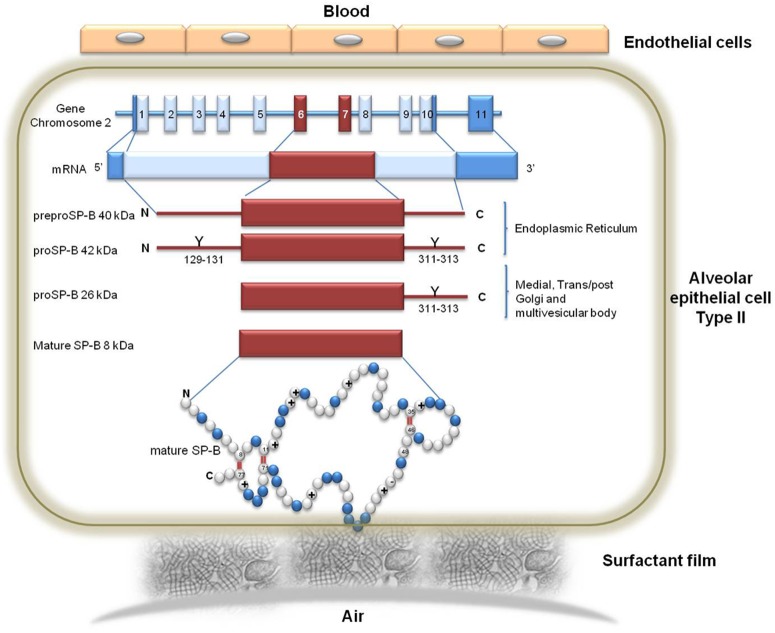
The surfactant protein type B (SPB) gene, mRNA and protein. The human SPB is encoded by 11 exons on chromosome 2. The SPB RNA of approximately 2 kb encodes a preprotein of 381 amino acids. Processing of the precursor includes removal of a signal peptide of approximately 23 residues, and glycosilatyion at amino acids 129 to 131 and 311 to 313. These events occur within the endoplasmic reticulum. Sequential proteolytic cleavages by proteases ultimately yield the 8 kDa 79 amino acid active mature SPB, which is encoded in exons 6 and 7. These sequential cleavage occurs in the medial and trans/post Golgi, and finally in the multivescicular body. Mature SPB sequence contains 52% hydrophobic amino acids, 8 conserved positively-charges residues and 1 conserved negatively-charged residue. The primary structure also includes 7 cysteines, six of which are involved in the formation of the three intra-molecular disulphide bridges, while the seventh cysteine is involved in an intermolecular disulphide responsible for the dimerization of the protein.

In the present setting of HF patients, the strongest correlation with DLCO was observed in the immature forms of SP-B. A few reasons are likely responsible of this finding. First, SP-B is produced only in the alveolar cells while SP-A and SP-D are also present in extra-pulmonary tissues including the trachea, brain, testes, salivary glands, heart, prostate, kidney. [41] Furthermore, in vivo and in vitro studies provide compelling support for the SP-D and SP-A as mediators of various immune-cell functions [41]. On the other hand, SP-B, is strictly required for the assembly of pulmonary surfactant and its extracellular development to form stable surface-active films at the air-liquid alveolar interface, making the lack of SP-B incompatible with life. Therefore the hydrophobic surfactant SP-B is likely the most appropriate indicator for the origin and function of the surfactant. Second, the immature forms of SP-B are the ones that are, from a biological-metabolic point of view, the SPs most unlikely to be present in the blood in normal circumstances. Indeed, the maturation process of SP-B is a complex multi-step process performed completely inside the alveolar epithelial cell being the highest concentration of the mature forms located near the cell air surface where they are eventually released while the immature forms are located inside cell organelles (endoplasmic reticulum, Golgi and multivescicular bodies) and are released only in case of cell membranes damage (Fig. 3). Unfortunately, immature forms of SPs other than SP-B have never been measured and the maturation process, if any, are still not completely defined. Therefore we cannot say if the described maturation behavior pertains only to SP-B or it is shared by other forms of SPs. However, the different role of SP-B mature form from others SPs suggests that the maturation process of SP-B is peculiar as it is the possible role of immature SP-B as precise marker of alveolar capillary membrane damage.

In conclusion our data show that immature forms of SP-B in the blood are the most precise biological markers of functional alveolar capillary membrane dysfunction available at present in HF patients. The value of SP-B immature forms in the clinical setting needs to be assessed.

## Supporting Information

S1 Data PAZIENT ID-Identification number. HF-Heart failure patient (1 = yes, 0 = no). SPBIMMATURE-Immature SPB (AU). SPB-Surfactant protein B (ng/mL). SPA-Surfactant protein A (ng/mL). SPD-Surfactant protein D (ng/mL). RAGE-Plasma receptor for advanced glycation end products (pg/mL). AGE-Age (years). GENDER-(1 = male, 0 = females). IPERTENSION-(1 = yes, 0 = no). DMII-Diabetes type II (1 = yes, 0 = no). weight-(Kg). height-(cm). BMI-Body mass index (kg/m2). FUMO-smoke (1 = yes, 0 = no, ex). LVEF-Left ventricular ejection fraction (%). Vtd-TeleDiastolic Volume (mL). Vts-TeleSystolic Volume (mL). IM-Mitral insufficiency (1 = yes, 0 = no). severityIM-Mitral insufficiency severity (1–4). GradoIT-Tricuspidalic insufficiency severity. TAPSE-Tricuspid annular plane systolic excursion (mm). PAPs-Systolic Pulmonary artery Pressure (mmHg). Hb-Hemoglobin (mg/dL). Crea-Creatinine (mg/dL). Na-Sodium (mmol/L). K-Potassium (mmol/L). Uric acid-(mg/dL). BNP-brain natriuretic petide (pg/mL). VC-Vital Capacity (L). VC_P-Vital Capacity (% of predicted). FVC-forced vital capacity (L). FVC_P-forced vital capacity (%). FEV1-forced expiratory volume in 1 second (L). FEV1_A-forced expiratory volume in 1 second (L). FEV1FVC-FEV1/FVC ratio. Dladj_ass-carbon monoxide lung diffusing capacity (mL/mm Hg/min). Dladj_perc-carbon monoxide lung diffusing capacity (% of predicted). SBPBASAL-Systolic blood pressure (mmHg). DBPBASAL-Diastolic blood pressure (mmHg). HRBASAL-Heart Rate (bpm). LOAD-Load at exercise (watt). QRPICk-Respiratory exchange ratio at peak. sbpPEAK-Systolic blood pressure at peak exercise (mmHg). DBPPEAK-Diastolic blood pressure at peak exercise (mmHg). VO_2_ kg-Oxygen uptake at peak exercise (mL/min/Kg). VO2ATKG-Oxygen uptake at anaerobic threshold (mL/min/Kg). VEVCO2slope-Ventilatory efficiency. NYHA-New York Heart Association functional class. ISCHAEMIC_ETIOLOGY-(1 = yes, 0 = no). ICD-implantable cardioverter-defibrillator (1 = yes, 0 = no). CRT-cardiac resynchronization therapy (1 = yes, 0 = no). BBLOCK-Betablocker (1 = yes, 0 = no). ACEI-Ace inhibitor (1 = yes, 0 = no). AT1 BLOCK-Angiotensin converting enzyme inhibitors (1 = yes, 0 = no). ANTIALDOSTERONIC-angiotensin type 1 receptor blockers (1 = yes, 0 = no). DIURETIC-(1 = yes, 0 = no). AMIODARONE-(1 = yes, 0 = no). DIGOXIN-(1 = yes, 0 = no). ANTICOAGULANT-(1 = yes, 0 = no). ANTIPLATELET-(1 = yes, 0 = no).
(XLSX)
